# Application of plant specialized metabolites to modulate soil microbiota

**DOI:** 10.5511/plantbiotechnology.23.0227a

**Published:** 2023-06-25

**Authors:** Akifumi Sugiyama

**Affiliations:** 1 Research Institute for Sustainable Humanosphere, Kyoto University, Gokasho, Uji, Kyoto 611-0011, Japan

**Keywords:** flavonoids, microbiota, plant specialized metabolites, rhizosphere, saponin

## Abstract

Plant specialized metabolites (PSMs) are considerably diverse compounds with multifaceted roles in the adaptation of plants to various abiotic and biotic stresses. PSMs are frequently secreted into the rhizosphere, a small region around the roots, where they facilitate interactions between plants and soil microorganisms. PSMs shape the host-specific rhizosphere microbial communities that potentially influence plant growth and tolerance to adverse conditions. Plant mutants defective in PSM biosynthesis contribute to reveal the roles of each PSM in plant–microbiota interactions in the rhizosphere. Recently, various approaches have been used to directly supply PSMs to soil by in vitro methods or through addition in pots with plants. This review focuses on the feasibility of the direct PSM application methods to reveal rhizospheric plant–microbiota interactions and discusses the possibility of applying the knowledge gained to future engineering of rhizospheric traits.

## Introduction

**Figure 1:**
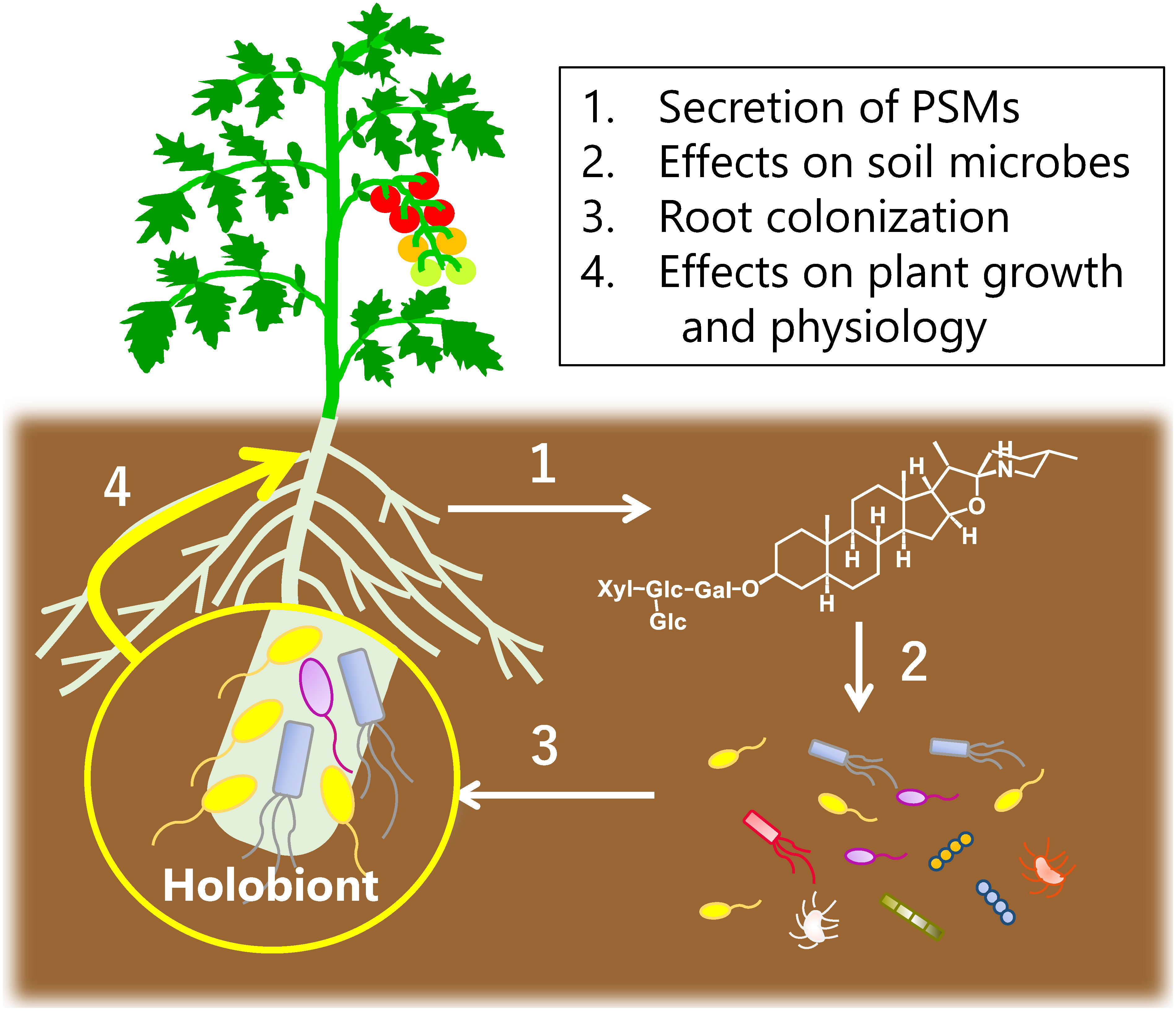
Figure 1. Model for plant specialized metabolite-mediated root microbiota formation and its effects on host plant.

Plant specialized metabolites (PSMs) play important roles in the adaptation of plants to both biotic and abiotic stresses. Plants store PSMs with antimicrobial activities in vacuoles and use them as chemical defense agents following infection with pathogenic microorganisms and attach by herbivores. Plants also use PSMs to establish beneficial relationships with other organisms. Symbiotic interactions with rhizobia and arbuscular mycorrhizal fungi are well-known examples, where PSMs such as flavonoids and strigolactones function as signaling compounds to initiate symbiosis (Zhang et al. 2015). The rhizosphere, which is defined as the soil region adjacent to plant roots (Hartmann et al. 2008), harbors a microbiome that exerts multiple effects on plant growth, fitness, and potential for crop production (Canto et al. 2020; Chialva et al. 2022). Recent studies have revealed the involvement of PSMs in shaping the rhizosphere microbiome by analyzing the root/rhizosphere microbiome of plant mutants with disruption in a particular biosynthetic pathway for PSMs (Jacoby et al. 2021; Pang et al. 2021; Pascale et al. 2020) (Figure 1). The loss-of-function approach is robust, although it lacks the ability to determine the effects of other metabolites in the rhizosphere, and both direct and indirect influence from plant mutant roots cannot be differentiated with this approach.

The application of pure chemicals to the soil is a promising method to directly assess the effect of PSMs on soil microbiomes. By repeated additions of PSMs to the soil, PSM concentration can be maintained at levels observed in the plant rhizosphere. Our group used the “pseudo-rhizosphere system” to reveal the effects of PSMs on shaping rhizospheric microbiota. In the present review, we summarize the functions of PSMs revealed by the methods for adding metabolites to the soil with or without plant growth and discuss the potential of the soil application of PSMs to engineer and regulate the rhizospheric environment to improve crop growth.

## An in vitro pseudo-rhizosphere system to analyze soil microbiota

**Figure 2:**
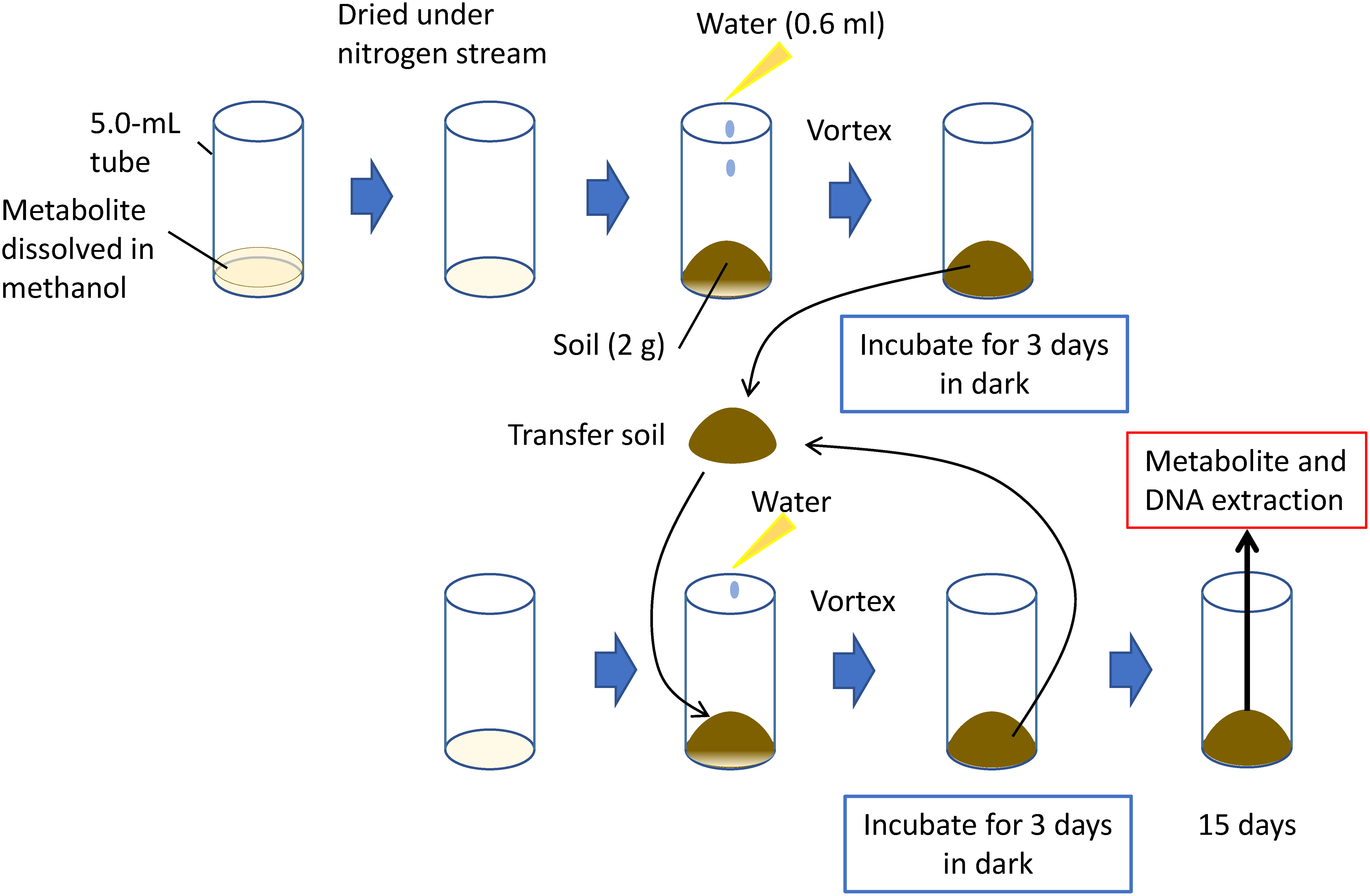
Figure 2. Schematic illustration of PSM treatment of soil in test tubes to achieve the pseudo-rhizosphere environment system.

We established an in vitro soil environment chemically similar to the rhizosphere by adding pure chemicals to the soil to analyze the effect of PSMs (Figure 2). Field soil was first air-dried and passed through a 2-mm sieve to remove stones, gravel, and roots. PSMs are often water-insoluble. We used organic solvents such as methanol to obtain a solution containing PSMs. Because organic solvents strongly influence microbial communities, these solvents must be removed before addition to the soil. We used methanol and dried solution containing PSM under nitrogen gas. Soil (2 g) was added to tubes containing dried metabolites, and sterilized water was added to obtain a water content ratio of 30%, which could be modified depending on the soil conditions of interest. The tubes were mixed thoroughly by using a vortex mixer and incubated in dark for 3 days. Because soil microorganisms degrade PSMs, we transferred the soil to another tube containing dried metabolites. This procedure was repeated 4 times, and after a 15-day incubation period, both DNA and metabolites were extracted for amplicon sequencing and liquid chromatography-mass spectrometry (LC-MS) analysis, respectively. Different concentrations of PSMs were to be used in this system to obtain a concentration of metabolites similar to that in the rhizosphere. This system can be easily manipulated in the laboratory and can be applied to any metabolites provided that pure chemicals are available. Microorganisms enriched in PSM-treated soil often possess ability to degrade it; this feature could be used to reveal the metabolic pathways of rhizospheric microorganisms. Bacterial strains with α-tomatine- and nicotine-metabolizing capability were isolated from soil treated with α-tomatine and nicotine, respectively (see below).

To comprehensively understand PSM-mediated interactions, it is essential to investigate their effects under various abiotic conditions. Abiotic factors such as pH, moisture content, temperature, and organic carbon and nitrogen contents affect the composition of microbiota (Bano et al. 2021); therefore, PMS-mediated interactions need to be investigated across a broad range of abiotic conditions. This pseudo-rhizosphere system favors the growth of aerobic bacteria. To assess the effects of PSM under anaerobic conditions that are likely present in the soil, the experiments need to be conducted under such conditions. Previous research has explored alterations in bacterial communities by PSMs under anaerobic conditions in gut microbiota (Ghimire et al. 2022; Huang et al. 2022; Li et al. 2022; Xu et al. 2020), which can help predict the effects of PSMs in anaerobic soil conditions. It is also crucial to accurately replicate the rhizosphere environment, which has a gradient of physical, chemical, and biological factors (Kuzyakov and Razavi 2019). Pseudo-rhizosphere systems utilizing artificial roots secreting metabolites have been utilized to study the effects of metabolites such as sugars, organic acids, and flavonoids in the rhizosphere (Buckley et al. 2022; Szoboszlay et al. 2016; Zhang et al. 2019). Artificial roots secreting PSMs in combination with primary metabolites including sugars and organic acids could be a more effective system to reproduce the rhizosphere plant–microbiota interactions. This is because primary metabolites have been shown to recruit plant growth-promoting rhizobacteria, although not exclusively (Upadhyay et al. 2022). Soil sampling at different points during the incubation will provide a comprehensive understanding of the spatiotemporal effects of PSMs in the rhizosphere. It is essential to note that not only microorganisms but also protists and nematodes can influence the rhizosphere microbiome, which should be considered when establishing the pseudo-rhizosphere system to further elucidate plant–microbiota interactions in the rhizosphere. Furthermore, sunlight, in particular far-red and near-infrared light, conducted in the root could impact root microbiota due to the phototaxis of soil microorganisms in field-grown plants (Losi and Gärtner 2021; Sun et al. 2004).

## Application of plant specialized metabolites to modify soil microbiota

### Flavonoids

Flavonoids, a group of phenolic compounds comprising more than 8,000 distinct molecules (Pietta 2000), protect plants from UV-B radiation, pathogens, and herbivores; attract pollinators; modify auxin transport and reactive oxygen species accumulation; and induce the *nod* genes of rhizobia for nodulation (Ferreyra et al. 2012; Weston and Mathesius 2013). Soybean [*Glycine max* (L.) Merrill] roots secrete isoflavones, daidzein and genistein, and the level of their secretion increases by approximately 10-fold under nitrogen-deficient conditions (Sugiyama et al. 2016). Under both hydroponics and field cultivation, the amount of secretion is higher during the early growth stage than during reproductive stages; however, daidzein levels in the rhizosphere are maintained throughout the growth stages because of its relatively slow degradation rate (half-life of ca. 7 days for daidzein) (Sugiyama et al. 2017; Toyofuku et al. 2021). Because isoflavone concentrations in soybean rhizosphere are maintained at sufficiently high levels to exhibit activity for microorganisms throughout the growth period, we tested whether isoflavones affect soil microorganisms other than rhizobia by creating a pseudo-rhizosphere environment in test tubes. The addition of daidzein to the soil in test tubes to obtain an equivalent amount of daidzein as that in soybean rhizosphere (10–20-nmol g^−1^ soil) decreased the α-diversity of bacterial communities as compared to that in the control soil; this was probably because of the inhibitory effects of isoflavones on soil microorganisms (Hassan and Mathesius 2012). Daidzein treatment increased 7 bacterial families and decreased 37 bacterial families as compared to those in the control soil; the bacterial communities of daidzein-treated soils became closer to that of soybean rhizosphere as compared to the bacterial communities of bulk soils based on the UniFrac distance, thus suggesting that daidzein functions in shaping the bacterial communities of soybean rhizosphere (Okutani et al. 2020). In particular, daidzein treatment enriched the abundance of Comamonadaceae, a major bacterial family in soybean rhizosphere, in a concentration-dependent manner. The members of Comamonadaceae showed growth promoting effects (Jiang et al. 2012; Sun et al. 2018); however, the functions of Comamonadaceae members enriched in soybean rhizosphere should be analyzed using the strains isolated from the rhizosphere. We isolated several bacterial species belonging to the family of Comamonadaceae from soybean rhizosphere, and the functional analyses of these isolates are currently in progress.

Treatment of the soil with flavonoid compounds induces changes in both bacterial and fungal communities (Sugiyama 2021). The application of isoflavone daidzein and genistein to the soil resulted in the formation of different microbial community structures (Guo et al. 2011). 7,4′-Dihydroxyflavone, a *nod* gene-inducing flavonoid of alfalfas (*Medicago sativa* L.), application modified bacterial communities and caused an increase in Acidobacteria; in contrast, naringenin application showed no significant alterations in soil bacterial communities (Szoboszlay et al. 2016). Luteolin application modified both bacterial and fungal communities of the rhizosphere soil of peanut (*Arachis hypogaea* L.) (Wang et al. 2018). In their study, luteolin application reduced growth and nodule formation in peanuts, suggesting the inhibitory effects of luteolin on the continuous cropping of peanuts. Maize (*Zea mays* L.) roots secrete flavones, apigenin and luteolin into the soil rhizosphere. A maize mutant (C2-Idf) defective in chalcone synthetase accumulates less flavones and exhibits symptoms of nitrogen deficiency when cultivated under nitrogen-deficient conditions. When this mutant was grown in a pot after growing the wild-type maize plant, the symptoms of nitrogen deficiency disappeared; on the other hand, when the wild-type plant was grown in a pot after the growth of the mutant, the wild-type plants exhibited nitrogen deficiency symptoms. These results suggest soil microbiota affected by the secreted flavones is involved in the adaptation to nitrogen deficiency conditions. Rhizosphere microbiota differed between the mutant and wild-type plants, with the reduction of Oxalobacteraceae in the mutant plant. Further characterization of the Oxalobacteraceae family members isolated from maize soil revealed that 9 of 16 isolates promoted the growth of C2-Idf mutants in nitrogen-poor soil. Treatment of the C2-Idf2 mutant with flavones had no effect on its growth under sterile condition; however, apigenin supplementation in unsterilized nitrogen-limiting soil restored the growth of the mutant. This finding suggests that the root-secreted apigenin increases Oxalobacteraceae members, which have growth-promoting effects on maize rhizosphere (Yu et al. 2021). The authors analyzed the effect of the microbiota shaped by flavones in the mutants defective in lateral root growth and demonstrated that the maize microbiota promotes lateral root formation under nitrogen-deficient conditions.

Flavonol addition also modifies microbiota. In the soil treated with quercetin, Proteobacteria was the most abundant family; furthermore, and at the amplicon sequence variant (ASV) level, ASVs of *Sphingobium* belonging to the family Sphingomonadaceae and ASVs of *Massilia* belonging to the family Oxalobacteraceae were enriched (Schütz et al. 2021). Eleven flavonoids secreted from soybean roots (daidzein, morin, coumesterol, genistein, naringenin, 7,4′-dihydroxyflavone, apigenin, isoliquiritigenin, luteolin, hesperetin, and chrysin) were added to the soil in the ratio secreted into the rhizosphere (Liu et al. 2021). In the flavonoid-treated soil, the members of Burkholderiaceae and Sphingobacteriaceae increased in soybean rhizosphere. As described earlier, flavonoids have various effects on rhizosphere microbiota; however, it was difficult to compare the results of various studies because of the difference in initial soil microbiota. A pseudo-rhizosphere system can be used to compare the effects of flavonoid treatments on soil with different microbial communities.

### Saponins

Saponins, a group compounds that are widely distributed in higher plants, consist of a hydrophobic backbone bound to hydrophilic saccharides, which leads to amphiphilicity (Vincken et al. 2007). Saponins are typically classified into triterpenoid saponins and steroid glycosides on the basis of the aglycone skeleton (Vincken et al. 2007). The accumulation of saponins in plants offers protection against pathogens and herbivores; saponins also function as allelochemicals that suppress the germination and growth of other plants. α-Tomatine is a steroidal glycoalkaloid that accumulates in leaves, roots, flowers, and green fruits of tomato (*Solanum lycopersicum* L.), and it is also secreted from the roots into the rhizosphere (Nakayasu et al. 2022). We used the pseudo-rhizosphere system to analyze the function of α-tomatine in modifying soil microbiota. α-Tomatine and tomatidine addition to the field soil significantly altered the bacterial communities in a concentration-dependent manner, with a decline in α-diversity probably because of the biological activities of α-tomatine and tomatidine. Treatment with α-tomatine and tomatidine decreased the relative abundance of 35 and 78 bacterial families, respectively, while it increased the members of the family Sphingomonadaceae (Nakayasu et al. 2021a). Sphingomonadaceae is the only family to be commonly increased in tomato rhizosphere and in soil treated with α-tomatine and tomatidine. Bacterial communities of α-tomatine- and tomatidine-treated soils were closer to those of the tomato rhizosphere soil as compared to those of bulk soil; this finding suggests that these metabolites secreted from tomato roots function in shaping the bacterial communities of tomato rhizosphere. Tomato *jre4-1* mutant is defective in jasmonate-responsive element (JRE) 4, a master regulator of the tomatine biosynthetic pathway; this mutant accumulates and secretes less α-tomatine (Nakayasu et al. 2021a, 2018). In the root and rhizosphere of the *jre4-1* mutant, the relative abundance of Sphingomonadaceae was decreased as compared to that in the wild-type plant; this finding further supported the functions of α-tomatine in enriching Sphingomonadaceae. Characterization of bacterial communities enriched in these soils revealed that a single ASV dominated in α-tomatine- and tomatidine-treated soil and in tomato rhizosphere. We previously isolated a strain of *Sphingobium* that has this sequence and can degrade α-tomatine in vitro (Nakayasu et al. 2021a). Further analysis of this isolate would reveal the metabolic pathway of α-tomatine in the rhizosphere and its involvement in tomato–microbiota interactions.

The application of other saponins also enriches Sphingomonadaceae in the pseudo-rhizosphere system. Soyasaponin Bb, a major soyasaponin found in the soybean rhizosphere, enriches the members of Sphingomonadaceae (Fujimatsu et al. 2020). At the genus level, *Novosphingobium* is increased in the soyasaponin Bb-treated soil, while *Sphingobium* is increased in α-tomatine-treated soil; these findings are consistent with the root-associated bacterial communities of soybean and tomato, where *Novosphingobium* and *Sphingobium* are more abundant than the other genera, respectively. These saponins have a common precursor, namely 2,3-oxidosqualene, which is biosynthesized through the mevalonate pathway; however, their backbone aglycone structures are different, i.e., α-tomatine is a steroidal glycoalkaloid, while soyasaponin is an oleanane-type triterpenoid saponin. To investigate whether this difference in backbone structure influences the abundance of Sphingomonadaceae in vitro, glycyrrhizin, an oleanane-type triterpenoid saponin, and the steroid saponins α-solanine and dioscin were added to the field soil in test tubes. As expected, glycyrrhizin enriched *Novosphingobium*, while α-solanine and dioscin enriched *Sphingobium*, suggesting a correlation between the chemical structure of the saponin backbone and the ability to alter bacterial communities (Nakayasu et al. 2021b). The application of ginsenosides (ginsenoside Rg1, Rb1, and Rh1) alters soil fungal communities, with an increment in pathogenic *Fusarium oxysporum* in ginsenoside-treated soils regardless of the structures tested (Li et al. 2020); however, the effects of ginsenosides on bacterial communities have not yet been tested. The application of ginsenoside Rg1 in combination with cellobiose and galacturonic acid also altered both bacterial and fungal communities and aggravated root rot disease caused by soil-born pathogen *Ilyonectria destructans*, which can metabolize ginsenoside Rg1 as a carbon source (Xu et al. 2021). Ginsenosides include both oleanane-type and dammarane-type triterpenoid saponins. It would be particularly interesting to determine whether these saponins differentially affect the rhizosphere microbial communities of *Panax* spp.

Although they are not classified as saponins, structural differences in cucurbitane-type triterpenoids also affect the PSM-mediated effects on soil rhizosphere. Cucurbitacins are a group of tetracyclic triterpenes that are mainly produced in Cucurbitaceae members such as cucumber (*Cucumis sativus* L.), melon (*Cucumis melo* L.), and watermelon [*Citrullus lanatus* (Thunb.) Mansfeld] (Hu et al. 2020). Cucurbitacin B, C, and E are found in melon and watermelon. Following its application to the soil of the melon plant, cucurbitacin B altered rhizosphere bacterial communities and suppressed the disease caused by *F. oxysporum*; in contrast, cucurbitacin C and E did not show any effects on disease suppression (Zhong et al. 2022). Cucurbitacin B probably enhances the growth of *Enterobacter*, leading to the colonization of *Bacillus* strains that are antagonistic to *F. oxysporum*. Subtle differences in the structure of cucurbitacins may affect the metabolic capacity of *Enterobacter* in the melon rhizosphere.

### Nitrogen-containing plant specialized metabolites

Plant roots secrete nitrogen-containing PSMs into the rhizosphere. Nicotine is an alkaloid found in *Nicotiana* spp. and plays a major role in defense against attack by herbivores and insects. In *Nicotiana tabacum*, nicotine is synthesized in roots and is transported into the leaf vacuole. Although nicotine is secreted from the roots, its role in the rhizosphere remains unknown. We used the pseudo-rhizosphere system to investigate the functions of nicotine in soil. Nicotine treatment altered the bacterial communities, enriching *Arthrobacter* belonging to the family Micrococcaceae, and leading to bacterial community compositions more similar to those of the tobacco endosphere than to those of the bulk soil (Shimasaki et al. 2021). During the evolution of the *Nicotiana* genus, some *Nicotiana* species acquired genes encoding the enzyme for synthesizing santhopine, an Amadori compound composed of fructose and glutamine, possibly through a horizontal gene transfer event from *Rhizobium* species (Quispe-Huamanquispe et al. 2017; Suzuki et al. 2002). Interestingly, santhopine also enriched *Arthrobacter* following its addition to the field soil. Both santhopine and dual-metabolite treatment of santhopine and nicotine modified the soil bacterial community, thereby forming a bacterial community closer to that of the tobacco endosphere than to that of the bulk soil. We isolated *Arthrobacter* strains from tobacco roots and metabolite-treated soil and sequenced their genomes. Almost all strains isolated from tobacco roots harbor genes to degrade nicotine and santhopine and exhibited catabolic activities for these metabolites in vitro; this finding suggests that the presence of genes to catabolize PSMs confers competence in rhizosphere environments rich in plant-specific PSMs (Shimasaki et al. 2021). *Arthrobacter* is a relatively abundant bacterial genus in tobacco roots; however, its roles in tobacco rhizosphere are yet to be revealed.

Benzoxazinoids are indole compounds biosynthesized in gramineous plants such as maize, wheat (*Triticum* spp.), and rye (*Secale cereal* L.), and they function as protective compounds against pathogens and insects (Zhou et al. 2018). Benzoxazinoids are secreted into the rhizosphere and modify the rhizosphere microbial communities that potentially protect plants from insects (Hu et al. 2018). Benzoxazinoid application alters soil microbiota. Following the addition of 2,4-dihydroxy-7-methoxy-2*H*-1,4-benzoxazin-3(4*H*)-one (DIMBOA) or MBOA to soil collected from a wheat field, these compounds were completely degraded within 4 days, suggesting active microbial degradation. Phospholipid-derived fatty acid analysis showed that benzoxazinoid application shaped different microbial communities in each soil (Chen et al. 2010). BOA-supplemented soil has a characteristic bacterial community with an increment in the relative abundances of Actinobacteriota and a decrease in Proteobacteria and Chloroflexi (Schütz et al. 2021). *Paenarthrobacter* and other bacterial species belonging to Actinobacteriota were isolated from BOA-treated soil; however, it remains to be determined whether these isolated species can metabolize BOA or affect plant growth.

Gramine, an indole compound, is a major allelochemical in barley (*Hordeum vulgare* L.). Both benzoxazinoids and gramine are derived from the tryptophan biosynthetic pathway; however, in barley, these biosynthetic pathways are mutually exclusive, and both are not biosynthesized in the same species of barley (Grün et al. 2005). Both benzoxazinoids and gramine function as protective compounds against a wide range of pathogens and insects; however, they have different effects on microbiota following their application to soil. Soil microbiota of gramine-treated soil is different from that of control and BOA-treated soil; this finding suggests that these nitrogen-containing PSMs exert differential effects on soil microbiota (Schütz et al. 2021). Modern cultivated varieties of barley frequently do not synthesize gramine (Maver et al. 2020). In another study, gramine was exogenously provided during the growth of barley cultivars that do not biosynthesize gramine, and their bacterial communities were analyzed (Maver et al. 2021). Gramine-treated barley varieties showed different bacterial communities than controls with an increase in 16 orders belonging to Acidobacteria, Actinobacteria, Bacteroidetes and Proteobacteria in a dose- and genotype-dependent manner. *Candidatus* genus *Nitrosotalea*, which is potentially involved in nitrification, was enriched in the rhizosphere of gramine-treated plants. It would be particularly interesting to determine whether gramine affects the nitrification process of soil microbiota as observed for sorgoleone, a root exudate secreted from sorghum [*Sorghum bicolor* (L.) Moench], which inhibits biological nitrification and modulates the rhizosphere bacterial communities of sorghum (Wang et al. 2021). Domestication of plant species caused a strong decrease in the genetic diversity of modern crops, which may have impacted the ability of plants to establish beneficial interactions with soil microorganisms (Pérez-Jaramillo et al. 2016). PSM-mediated interactions could be specific to particular cultivar or genotype, as some PSMs may have lost their functions during the domestication, similar to the case for gramine.

### Sulfur-containing plant specialized metabolites

Glucosinolates are sulfur-containing PSMs consisting of a β-D-thioglucose moiety connected to a sulfonated aldoxime and a side chain. They are produced by plants of the order Brassicales, which includes the model plant *Arabidopsis thaliana* (Blazevic et al. 2020; Halkier and Gershenzon 2006). Myrosinase, the enzyme responsible for the hydrolysis of glucosinolates, and glucosinolates are sequestered in separate cellular compartments. Following cell disruption by pathogen attack, myrosinase mixes with glucosinolates. Myrosinase-catalyzed hydrolysis of the thioglucoside linkage results in the formation of glucose and an unstable aglycone that can be rearranged to form isothiocyanates, which are toxic metabolites to a wide range of soil-borne pests and pathogens (Ntalli and Caboni 2017). 2-Phenylethyl isothiocyanate application to a luvisol soil affected the soil bacterial community as revealed by denaturing gradient gel electrophoresis of 16S rDNA (Rumberger and Marschner 2003), while the other group showed no effect of 2-phenylethyl isothiocyanate on the alteration of soil bacterial communities (Omirou et al. 2011). The application of different types of isothiocyanates, including 2-propenyl isothiocyanate, butyl isothiocyanate, phenyl isothiocyanate, and benzyl isothiocyanate, altered soil bacterial and fungal communities. Varying effects on the microbiome were observed according to the structure of isothiocyanates. 2-Propenyl isothiocyanate exerted a stronger influence on both bacterial and fungal communities than other isothiocyanates (Hu et al. 2015). Rapeseed extract mainly containing 2-hydroxy-3-butenyl-glycosinolate (progoitrin) and 3-butenyl glucosinolate (gluconapin) was incubated with myrosinase to convert glucosinolates to isothiocyanates (Siebers et al. 2018). Myrosinase-treated rapeseed extract containing goitrin, which is derived from spontaneous cyclization of 2-hydroxy-3-butenyl isothiocyanate, as a major product altered both bacterial and fungal communities after its application to the soil. Bacterial taxa of Gammaproteobacteria, Bacteriodetes, and Acidobacteria and fungal taxa of *Trichosporon* were enriched (Siebers et al. 2018). These differential effects on soil microbiota suggest that the effects of isothiocyanates on microbial communities probably depend on isothiocyanate structure, treatment method, and soil characteristics.

Glucosinolates are not necessarily degraded only by plant myrosinase; following their secretion into the rhizosphere, microbial myrosinase degrades glucosinolates to form isothiocyanates and other breakdown products. Hanschen et al. investigated the effects of the application of pure 2-propenyl glucosinolates with or without myrosinase on the bacterial community composition (Hanschen et al. 2015). Propenyl glucosinolate (sinigrin) addition without myrosinase had a higher effect on the bacterial communities than 2-propenyl glucosinolate application with myrosinase; this finding suggests that glucosinolates have a distinctive influence on soil microbiota than isothiocyanates. This difference could be due to an increase in the ability of bacteria to utilize glucosinolates as a carbon source and a decrease in bacterial vulnerability to toxic degradation products.

## Conclusion and future perspectives

The rhizosphere microbiome plays a key role in plant growth and tolerance to various stresses (Carrion et al. 2019; Kwak et al. 2018); therefore, engineering the rhizosphere microbiome has high potential to achieve sustainable agriculture to support the growing demand of foods and alleviate negative effects on environment (Ke et al. 2021). The inoculation of plant growth-promoting bacteria and fungi into soil rhizosphere has been conducted in both laboratories and fields for decades. Although microbial inoculants have been commercialized to improve plant health and crop yield, impediments for their long-term success in agriculture lay in root colonization, persistence in the rhizosphere, and consistent responses under different soil and climatic conditions (Rilling et al. 2019). Engineering the rhizosphere metabolites is a propitious solution to overcome the limited root colonization by microorganisms. This review summarized recent findings on PSM-mediated alterations in soil microbiome with a special focus on the direct application of metabolites (Table 1). Transgenic *Arabidopsis* expressing the gene to synthesize octopine, an opine released from crown gall tumors, secretes octopine into the rhizosphere and favors the growth of *Ensifer* that can utilize octopine as a carbon source (Mondy et al. 2014). Furthermore, transgenic *Medicago truncatula* and barley expressing genes for producing rhizopine, a scylloinosamine, harbor *Rhizobia* that can utilize rhizopine (Geddes et al. 2019). In addition to the modification of host plant trait to secrete PSMs, the direct application of PSM in the soil can alter the rhizosphere metabolome, thereby favoring the growth and colonization of microorganisms attracted to and/or capable of utilizing PSMs. The enriched microorganisms often possess metabolic pathways that allow them to tolerate the inhibitory and adverse effects of the PSMs (Nakayasu et al. 2021a; Shimasaki et al. 2021); however, it is not yet clear whether enriched microorganisms exert a beneficial or harmful influence on host plants. It is crucial to test the effects of exogenously applied PSMs on root colonization by microorganisms and their influence on plant growth. When using PSMs for field-grown plants, possibility of toxicity to soil microorganisms and environment should also be considered, similar to the risks associated with agrochemicals such as pesticides (Karpouzas et al. 2022). Additionally, the manipulation of microbial genomes to improve the capability of the strain to utilize PSMs as a signaling or carbon source could enhance the ability of transgenic microorganisms to colonize host roots efficiently while competing with indigenous soil microorganisms.

**Table 1:** Table 1. Chemical structures of plant specialized metabolites and analysis of their effects on soil microbiota through the addition of pure chemicals.

Group	Compound	Chemical structure	Treatment	Effects on microbiota	References
Flavonoids	Apigenin	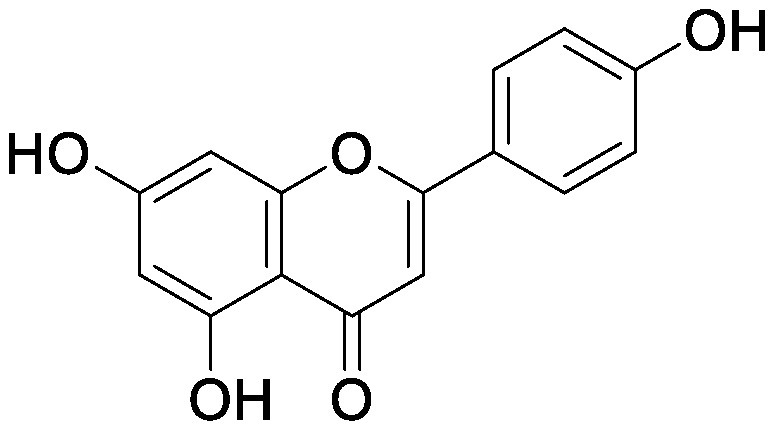	Maize grown in pot	•Altered soil bacterial microbiome •Growth promotion under nitrogen deficiency	Yu et al. (2021)
	Daidzein	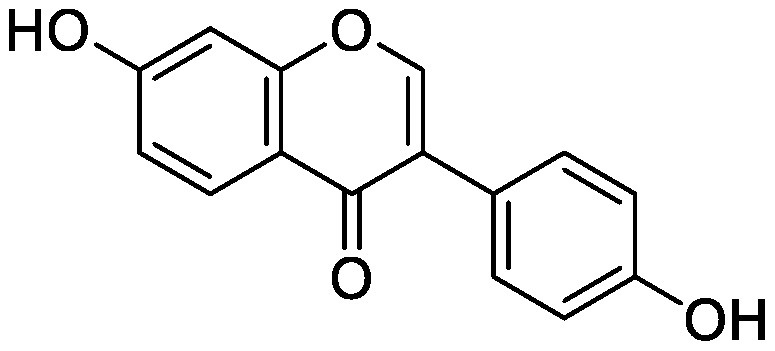	Field soil in a vial	•Altered soil microbiome	Guo et al. (2011)
			Field soil in a test tube	•Altered soil bacterial microbiome closer to that of soybean rhizosphere than that of bulk soil •Enrichment of Comamonadaceae	Okutani et al. (2020)
	7,4′-Dihydroxyflavone	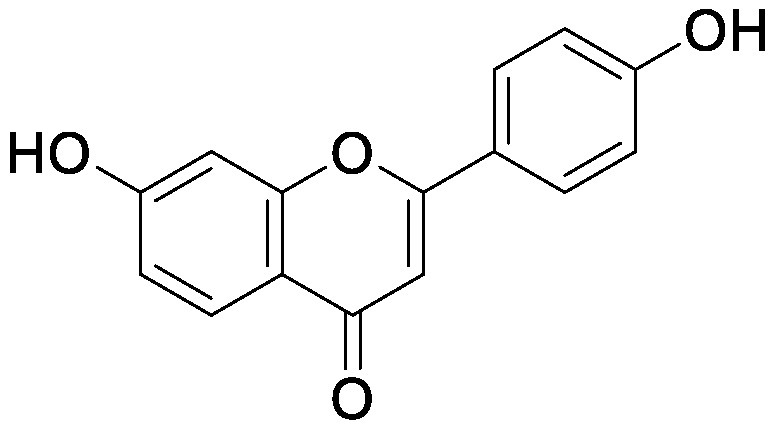	Field soil in a conical tube	•Altered soil bacterial microbiome •Enrichment of Acidobacteria	Szoboszlay et al. (2016)
	Flavonoid mixture		Soybean grown in a pot	•Altered soil bacterial microbiome •Enrichment of Burkholderiaceae, Methylobacteriaceae, and Sphingobacteriaceae	Liu et al. (2021)
	Genistein	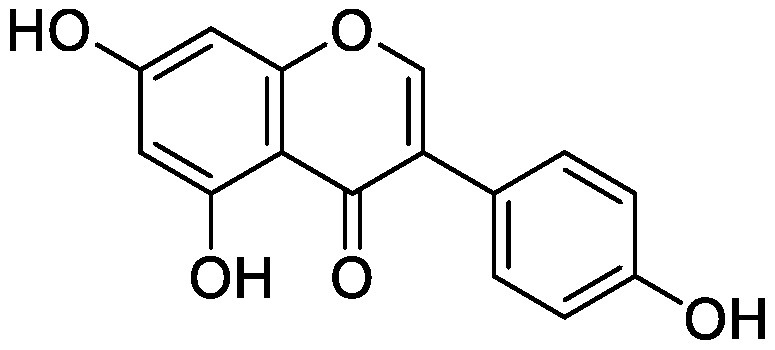	Field soil in a vial	•Altered soil microbiome	Guo et al. (2011)
	Luteolin	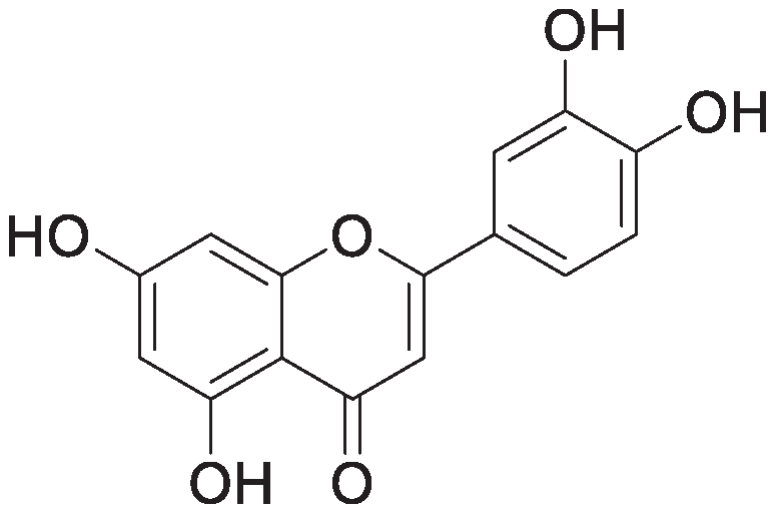	Peanuts grown in a pot	•Altered soil microbiome •Reduced nodulation	Wang et al. (2018)
	Quercetin	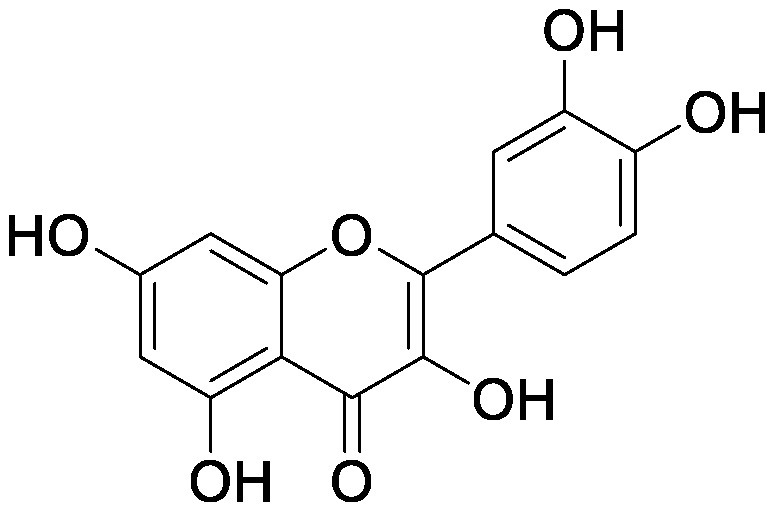	Field soil in a pot	•Altered soil bacterial microbiome •Enrichment of ASVs belonging to Proteobacteria	Schütz et al. (2021)
Triterpens	Cucurbitacin B	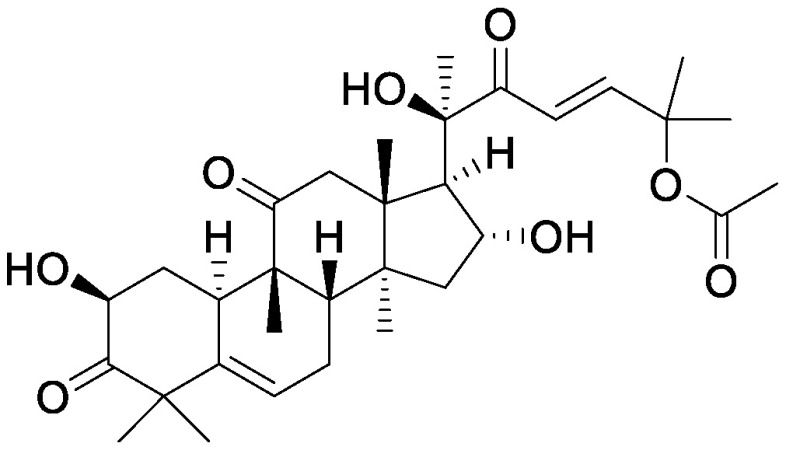	Melon grown in a pot	•Altered soil bacterial microbiome •Suppression of melon wilt disease caused by *Fusarium oxysporum*	Zhong et al. (2022)
Steroidal saponins	Dioscin	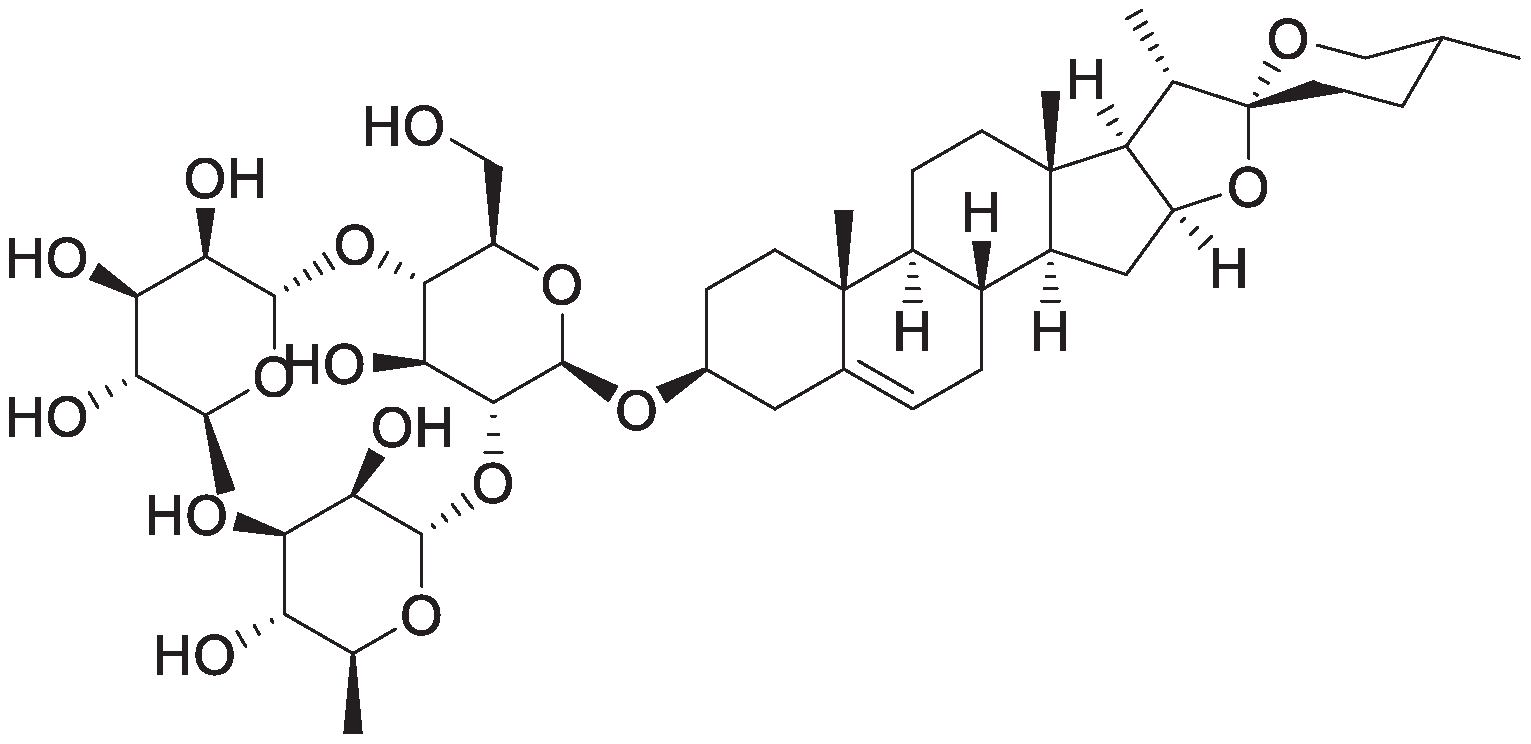	Field soil in a test tube	•Altered soil bacterial microbiome •Enrichment of *Sphingobium*	Nakayasu et al. (2021b)
	α-Solanine	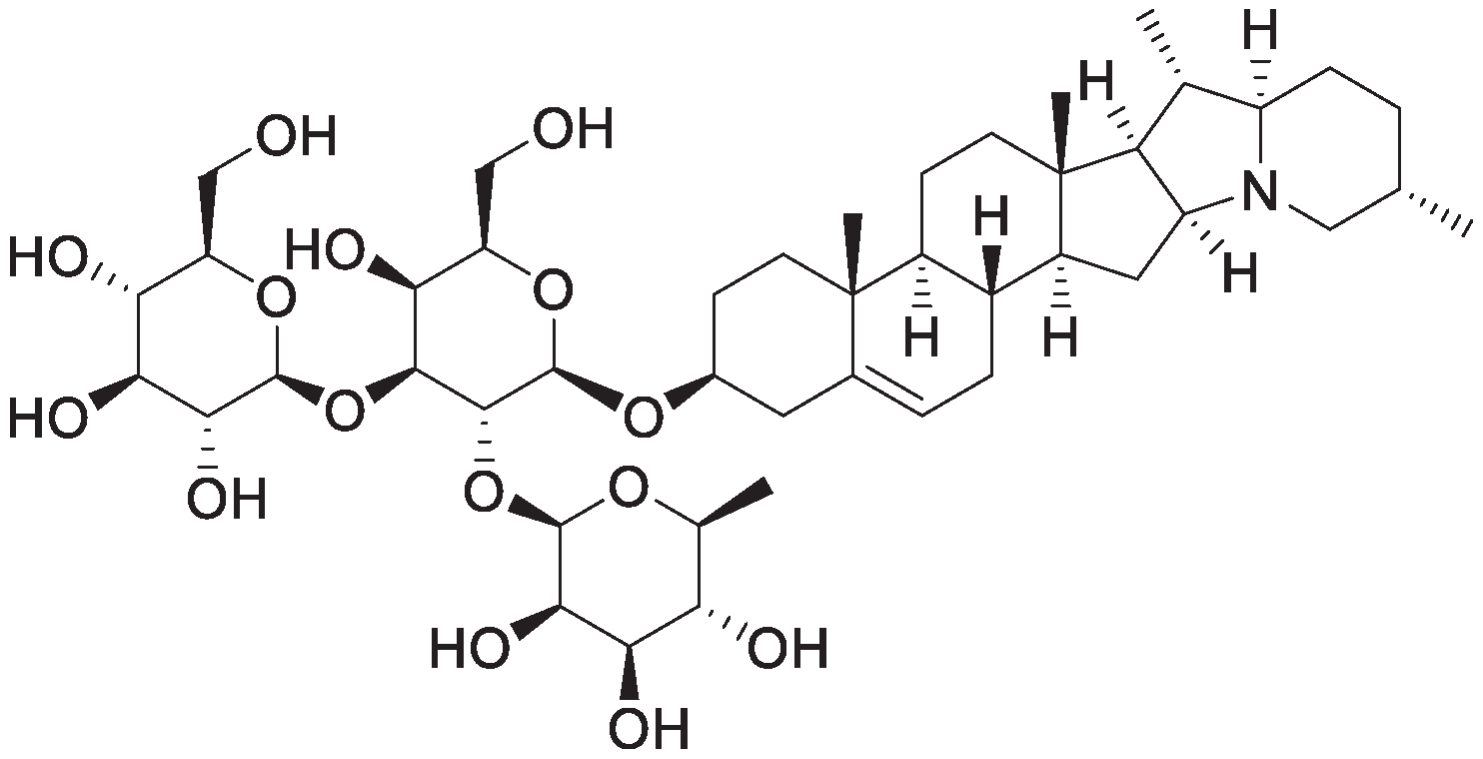	Field soil in a test tube	•Altered soil bacterial microbiome •Enrichment of *Sphingobium*	Nakayasu et al. (2021b)
	α-Tomatine	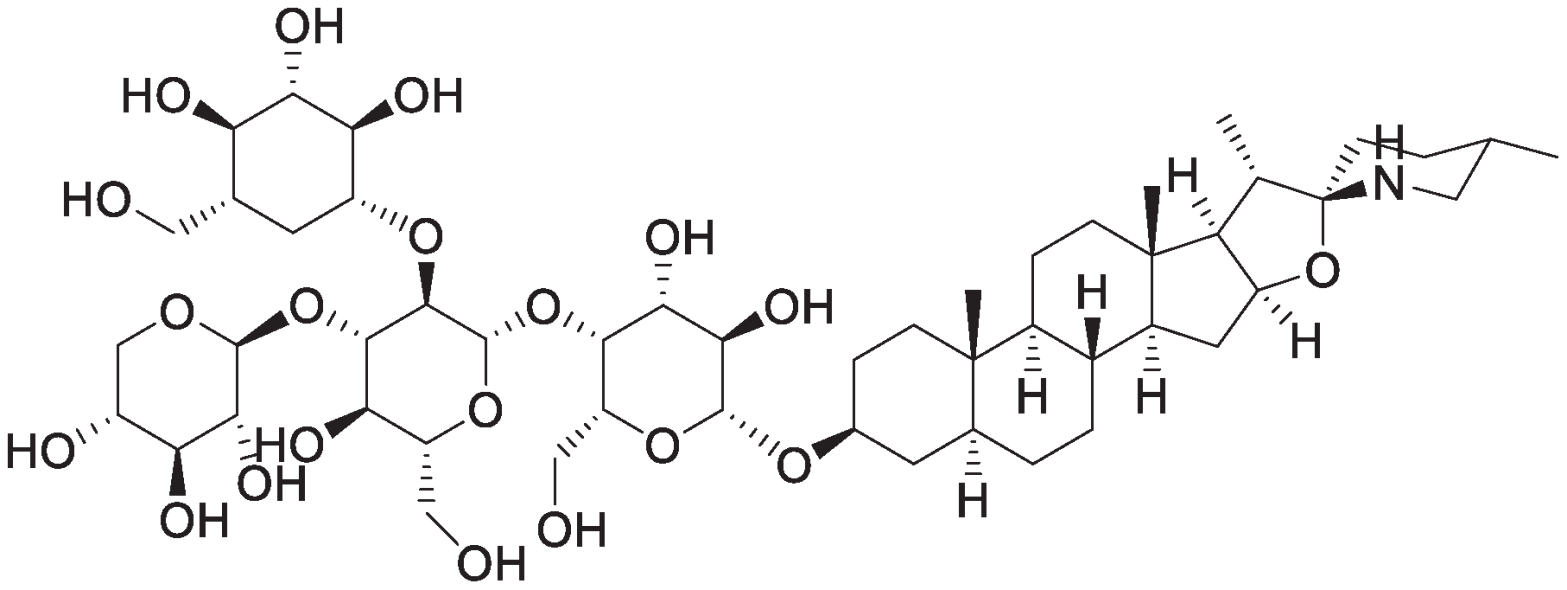	Field soil in a test tube	•Altered soil bacterial microbiome closer to that of tomato rhizosphere than to that of bulk soil •Enrichment of *Sphingobium*	Nakayasu et al. (2021a)
Triterpenoid saponins	Ginsenoside Rg1	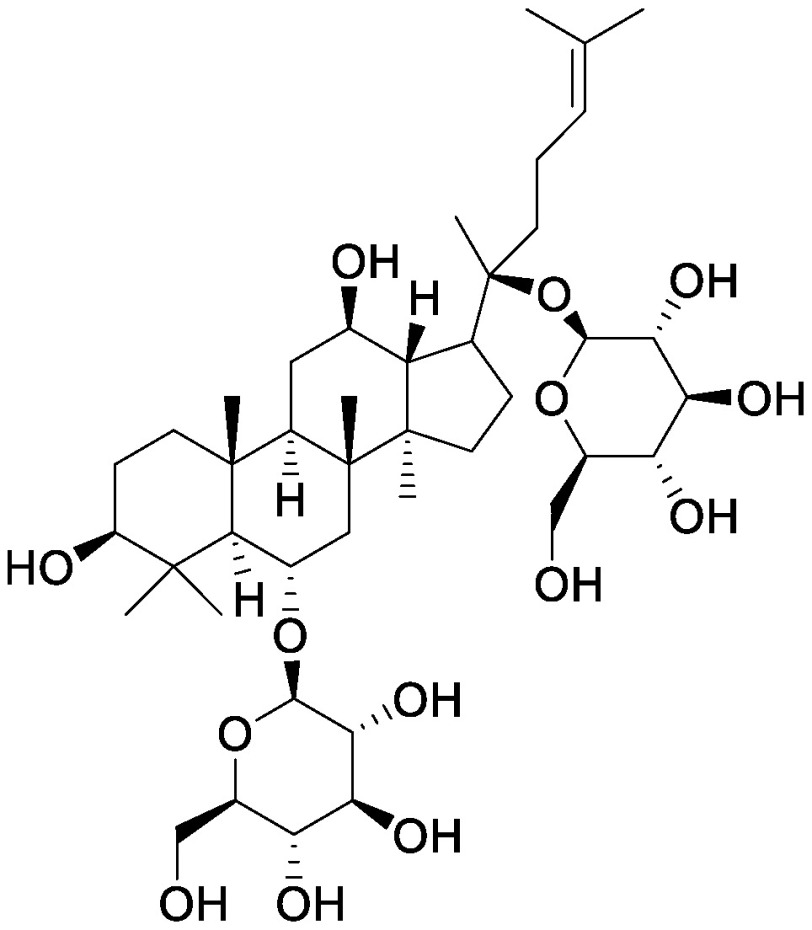	Hillside soil in a bottle	•Altered soil fungal microbiome •Increase in *Fusarium oxysporum*	Li et al. (2020)
			Natural and conditioned soil in a pot	•Altered soil bacterial and fungal microbiome	Xu et al. (2021)
	Glycyrrhizin	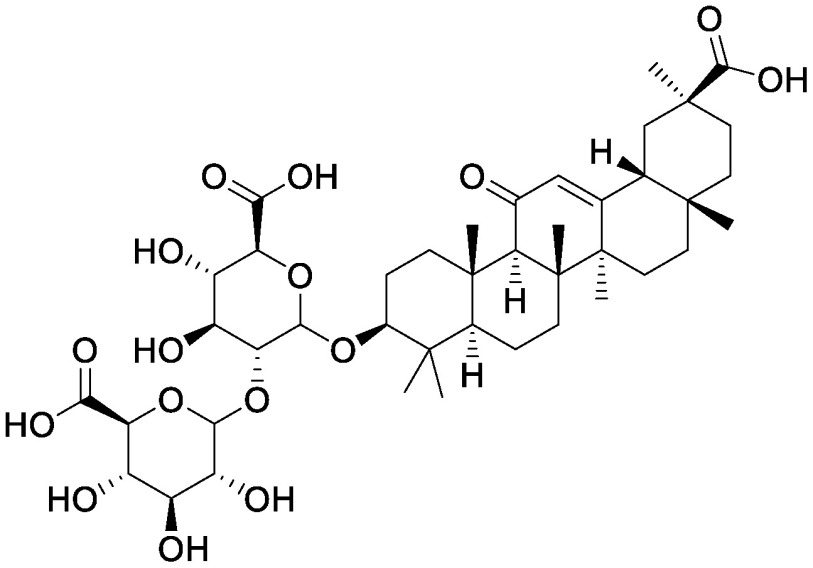	Field soil in a test tube	•Altered soil bacterial microbiome •Enrichment of *Novosphingobium*	Nakayasu et al. (2021b)
	Soyasaponin Bb	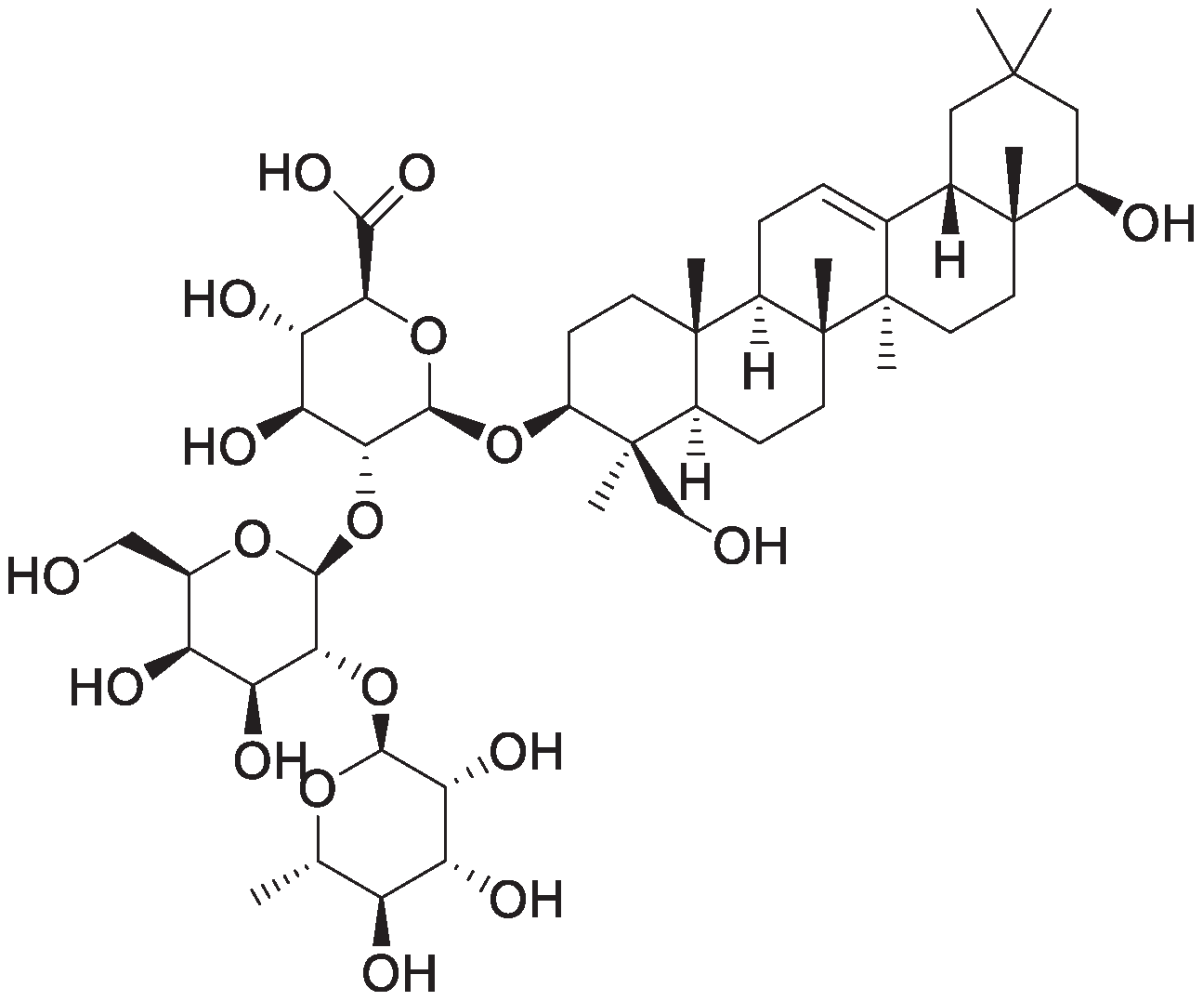	Field soil in a test tube	•Altered soil bacterial microbiome •Enrichment of *Novosphingobium*	Fujimatsu et al. (2020)
Alkaloids	Gramine	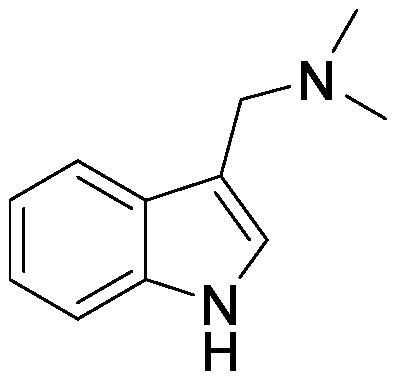	Barley grown in a pot	•Altered soil bacterial microbiome •Enrichment of Nitrosotaleales	Maver et al. (2021)
			Field soil in a pot	•Altered soil bacterial microbiome •Enrichment of ASVs belonging to Proteobacteria	Schütz et al. (2021)
	Nicotine	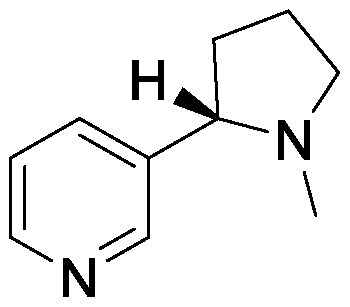	Field soil in a test tube	•Altered soil bacterial microbiome closer to that of tobacco endosphere than to that of bulk soil •Enrichment of *Arthrobacter*	Shimasaki et al. (2021)
Benzoxazinoids	BOA	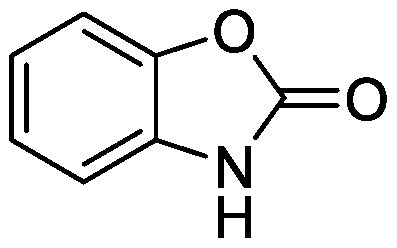	Field soil in a pot	•Altered soil bacterial microbiome •Enrichment of ASVs belonging to Actinobacteriota	Schütz et al. (2021)
	DIMBOA	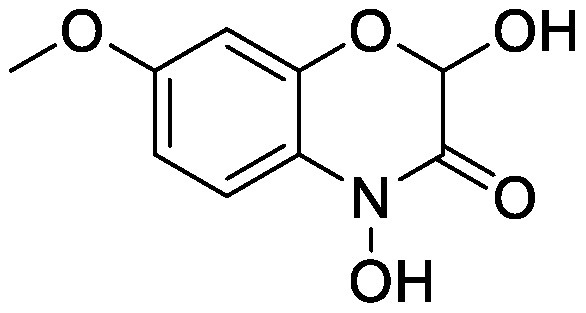	Field soil in a vial	•Altered soil microbiome	Chen et al. (2010)
Glucosinolates	Sinigrin	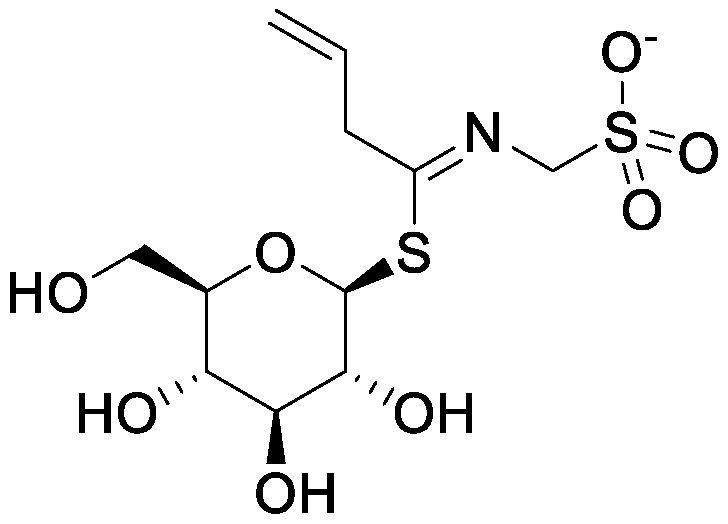	Soil from tree nurseries in a glass vial	•Altered soil bacterial microbiome	Hanschen et al. (2015)
Isothiocyanates	2-Phenylethyl isothiocyanate	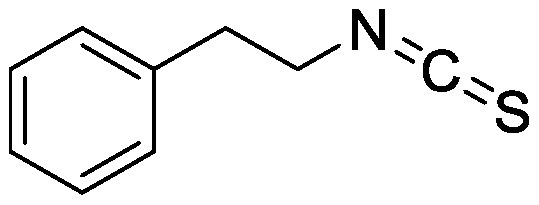	Luvisol soil	•Altered soil bacterial microbiome	Rumberger and Marschner (2003)
	2-Propenyl isothiocyanate	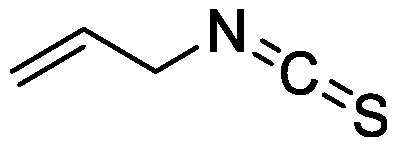	Field soil in a container	•Altered soil bacterial and fungal microbiome	Hu et al. (2015)
	Rapeseed extract treated with myrosinase (goitrin)	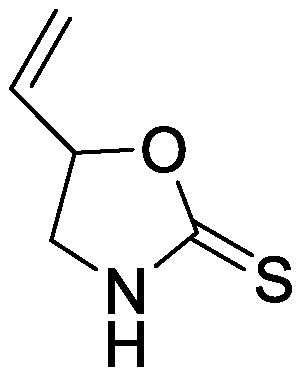	Field soil in a pot	•Altered soil bacterial and fungal microbiome •Enrichment of *Trichosporon*	Siebers et al. (2018)
Others	Santhopine	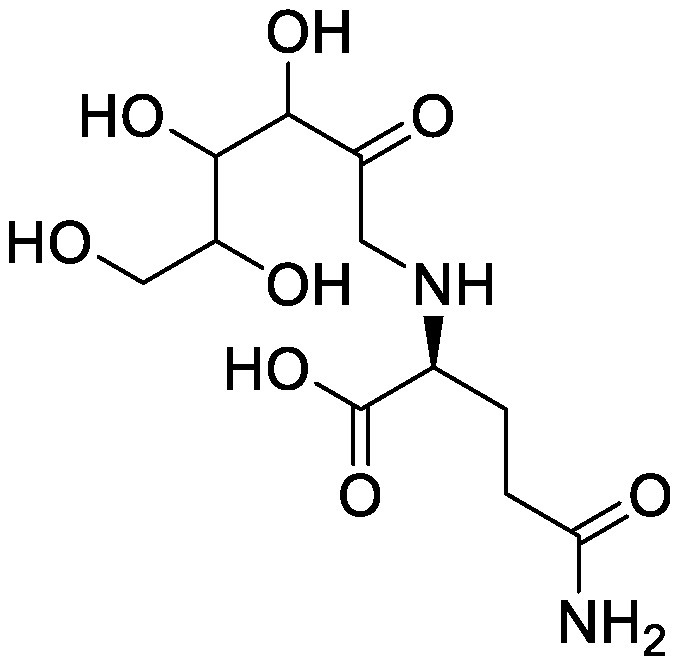	Field soil in a test tube	•Altered soil microbiome closer to that of tobacco endosphere than to that of bulk soil •Enrichment of *Arthrobacter*	Shimasaki et al. (2021)

**Figure 3:**
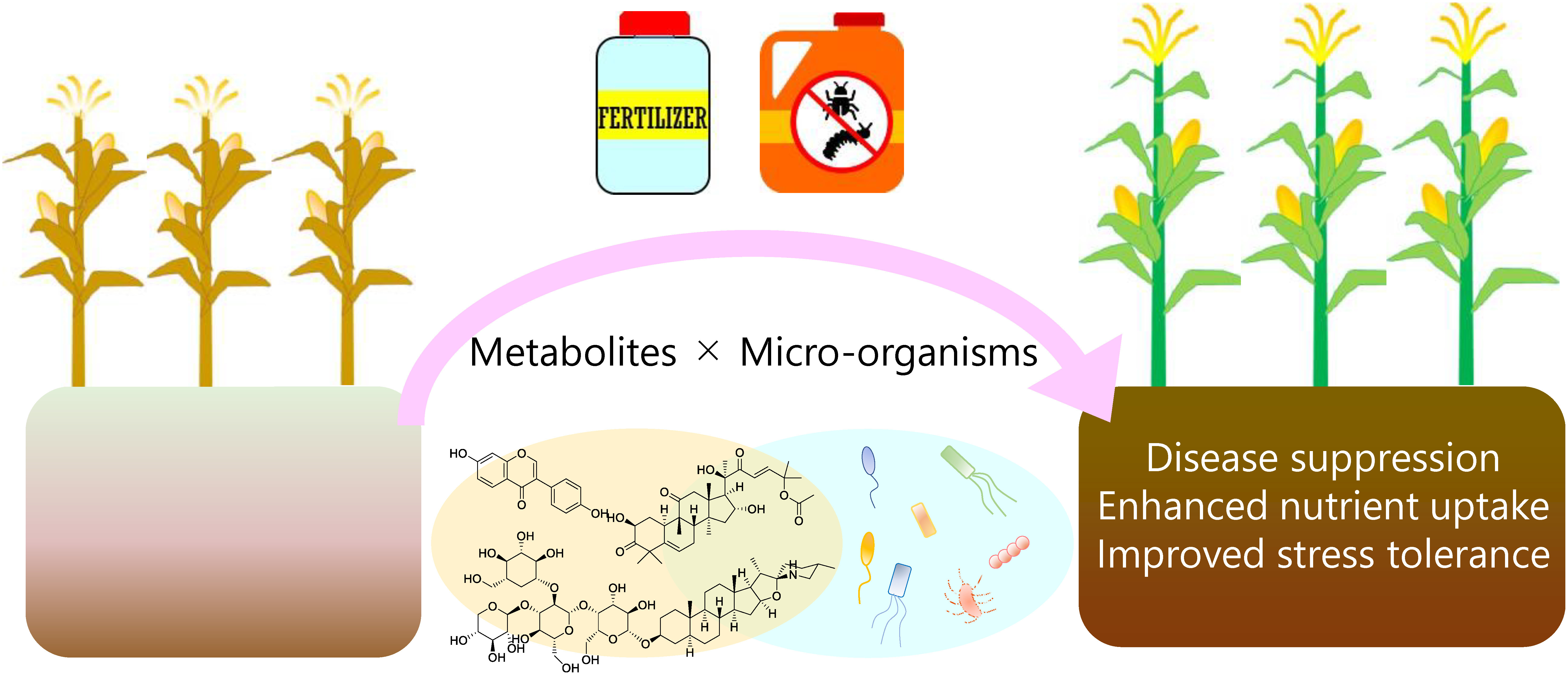
Figure 3. Integration of metabolites and microorganisms to engineer the rhizosphere for promoting plant growth and mitigating stress. The combination of plant growth-promoting microorganisms and plant specialized metabolites can promote the root colonization of microorganisms, leading to the establishment of “good” microbiome.

PSMs have been used as a biostimulant to alleviate the unfavorable effects of abiotic and biotic stresses such as drought, high temperature, and salinity, although their mechanisms of action remains largely unclear (Ben Mrid et al. 2021). Less focus was given to the microbiome in PSM-oriented research before the recent discoveries in PSM-mediated modification of microbiota. Because PSM-treatment to soil can modify the rhizosphere microbiota and improve plant growth under unfavorable conditions (Yu et al. 2021; Zhong et al. 2022), comprehensive understanding of PSM–microbiome–host plant communication is critical to develop approaches for PSM-mediated rhizosphere engineering for crop production. Host plant and its microbiota are regarded as a unique biological entity called holobiont, in which the host and microbiota interact with each other to affect the development and physiology within the holobiont (Hassani et al. 2018; Rosenberg and Zilber-Rosenberg 2016). PSMs could be key mediators in the holobiont. Practically, the combined application of PSMs with potentially plant growth-promoting cocktail of microorganisms to establish a holobiont would be a propitious solution to overcome the limited root colonization of soil microorganisms and improve the effectiveness of inoculants in the agricultural fields; this approach together with appropriate fertilizer and pesticide application could eventually enhance crop production (Figure 3).

## Abbreviations

ASV: amplicon sequence variant
PSM: plant specialized metabolite
